# Clinical outcomes and risk factor of immune checkpoint inhibitors-related pneumonitis in non-small cell lung cancer patients with chronic obstructive pulmonary disease

**DOI:** 10.1186/s12890-022-02190-w

**Published:** 2022-12-01

**Authors:** Zhu Zeng, Jingjing Qu, Yake Yao, Fei Xu, Shan Lu, Pei Zhang, Yinan Yao, Ning Li, Jianying Zhou, Yuehong Wang

**Affiliations:** 1 Department of Respiratory Diseases, Thoracic Disease Center, The First Affiliated Hospital, Zhejiang University School of Medicine, Hangzhou, Zhejiang China; 2 Department of Medical Oncology, The First Affiliated Hospital, Zhejiang University School of Medicine, Hangzhou, China; 3 Department of Respiratory Medicine, The First Affiliated Hospital, Zhejiang University School of Medicine, Qingchun Road 79, Hangzhou, China

**Keywords:** Checkpoint inhibitors related pneumonitis, Chronic obstructive pulmonary disease, Immune-related adverse events, Immune checkpoint inhibitors, Lung cancer

## Abstract

**Objectives:**

Chronic obstructive pulmonary disease (COPD) is the most common co-morbidity associated with non-small cell lung cancer (NSCLC) patients. Immune checkpoint inhibitors related pneumonitis (CIP) is a common immune-related adverse event that can be life-threatening. The study aims to evaluate the association of COPD with the incidence and outcome of CIP in NSCLC patients receiving immune checkpoint inhibitors (ICIs).

**Materials and methods:**

We retrospectively collected data from 122 patients diagnosed with NSCLC and treated with ICIs in our department. Baseline pulmonary function was performed in the whole cohort. The incidence, risk factors, treatment and outcome of CIP patients were evaluated. Furthermore, the efficacy of ICIs in patients with COPD was analyzed.

**Results:**

Nineteen patients (15.5%, 19/122) developed CIP during ICIs treatment, most patients with CIP were grade 1–2, and the incidence of CIP was comparable in patients with COPD and those without COPD (18.0% vs. 13.1%, *P* = 0.618). In addition, an increasing trend in the incidence of CIP among patients with pulmonary fibrosis on baseline chest CT scans (27.3% vs. 13.0%, *P* = 0.093). There is a longer progression-free survival in COPD patients than the non-COPD patients.

**Conclusion:**

Coexisting COPD did not predict the higher risk of CIP in NSCLC treated with ICIs therapy. Nevertheless, pre-existing pulmonary fibrosis on CT scan may increase the risk of CIP, close monitoring is advised in these patients during ICIs.

## Introduction

Lung cancer is the major cause of cancer-related deaths worldwide [1]. The anti-programmed cell death 1 (PD-1)/anti-programmed cell death-ligand 1 (PD-L1) immune checkpoint inhibitors (ICIs) block immune checkpoint pathways, activating a tumor specific T cell immune response [2]. Clinical trials have demonstrated that immunotherapy showed a significant clinical and survival benefit to advanced non-small cell lung cancer (NSCLC) patients [3, 4]. Clinical trials reported a higher quality of life and less treatment-related toxicity with ICIs than standard chemotherapy; however, ICIs have unique side effects in various organs, termed immune-related adverse events (irAEs). The irAE profiles vary from tumor types, and different immune microenvironments may drive histology-specific irAE patterns [5]. A previous study indicated that immune checkpoint inhibitors related pneumonitis (CIP) might occur more often and have a faster onset in NSCLC than in other types of cancer [6]. CIP is a life-threatening irAE that may result in wide-ranging respiratory symptoms with pulmonary parenchymal abnormalities and result in respiratory failure. Although a few research has been done, the risk factors of CIP remain inconclusive [6]. The assessment of the risk factors for CIP and other life-threatening irAEs is a growing clinical need in the effort to personalize and predict the side effects of cancer therapy. Previous studies have suggested that the presence of chronic inflammation prior to immunotherapy, such as rheumatoid arthritis, and lupus, can confer an increased risk of irAEs that involve the target organ of prior injury [7]. Pre-existing pulmonary diseases, including asthma, interstitial lung disease (ILD), pneumothorax and pleural effusion have been reported to be closely associated with the development of CIP in patients with NSCLC [8–11].

Chronic obstructive pulmonary disease (COPD) is a common comorbidity in lung cancer. COPD prevalence in newly-diagnosed lung cancer patients was estimated about 50%, and COPD is considered an independent risk factor of lung cancer [12, 13]. Increasing evidence supports that lung cancer patients with coexistence of COPD might respond better to immunotherapy, which can partially be attributed to the fact that the expression of immune checkpoint proteins PD-1 and PD-L1 is dysregulated in COPD patients [14]. However, the safety of ICIs in lung cancer patients with coexistence of COPD, especially the incidence and outcome of CIP is uncertain. Given the high prevalence of COPD in the lung cancer population and the clinical imperative to determine the comorbidities that might increase a patient’s risk of immune-related pneumonitis, we sought to investigate whether a prior diagnosis of COPD was associated with a higher incidence of CIP. Moreover, we also explored the correlations between clinical risk factors such as pulmonary computed tomography (CT) abnormalities (fibrosis/emphysema) at baseline and the incidence of CIP in lung cancer patients with COPD.

## Materials and methods

### Patients and study approval

Consecutive patients with histologically confirmed NSCLC who received PD-1 antibodies (nivolumab or pembrolizumab) as routine treatment at the First Affiliated Hospital, College of Medicine, Zhejiang University between December 1, 2018, and May 1, 2021 were reviewed. The last follow-up occurred on December 1, 2021. Patients who received at least 2 cycles of ICIs treatment and underwent chest CT in the 3 months before and after anti-PD-1 therapy were included in this study. Patients with a history of using prior ICIs, thoracic radiotherapy, previous history of autoimmune disease, a previous known history of ILD before diagnosis of lung cancer and required pharmacotherapies to alleviate the symptoms were excluded. The whole cohort was enrolled to analyze the correlation of COPD with the risk of CIP. Among the 122 patients, 21 patients received ICIs as neoadjuvant therapy (stage IIB and IIIA). Hence the overall survival (OS) and progression-free survival (PFS) of ICIs were evaluated in 101 stage IV NSCLC patients. Clinical and treatment information was obtained from our electronic medical records. This study was approved by the Ethics Committee of the First Affiliated Hospital, Zhejiang University School of Medicine (Registration No. IIT20220383A). Individual consent for this study was waived by the Ethics Committee of the First Affiliated Hospital, Zhejiang University School of Medicine as the privacy of the patients has not been disclosed. The study was conducted in accordance with the Declaration of Helsinki (as revised in 2013).

### Radiographic and pulmonary function analysis

Baseline chest CT scan findings and pulmonary function testing were recorded, the most recent chest CT scan obtained before the initiation of ICI treatment was recorded as a baseline. A retrospective radiology review of serial chest CT scans of all patients was independently performed by two respiratory physicians. The fibrosis (F) and emphysema (E) scores were evaluated according to the scoring system reported in previous studies [15–17]. The fibrosis score (F score, 0–5) was visually evaluated according to the interlobular septal thickening and discrete honeycombing area, while the emphysema score (E score, 0–4) was determined visually according to the diameter of low attenuation areas (Table 1). Pulmonary function measurements, including spirometry and diffusing capacity of the lung for carbon monoxide (DLco), were performed using respiratory analyzer (Quark PFT, COSMED, Rome, Italy). Diagnoses and classifications of COPD were made in accordance with the Global Initiative for Chronic Obstructive Lung Disease criteria [18], which COPD were defined as those having a forced expiratory volume in 1 s (FEV1) to forced vital capacity ratio (FVC) < 0.7 after bronchodilator use.

**Table 1:** Score systems of fibrosis and emphysema

	Fibrosis score(F score)	Emphysema score (E score)
0	No fibrosis	No low attenuation areas (LAAs)
1	Interlobular septal thickening; no discrete honeycombing	Sparse, scattered small LAAs up to 5 mm in diameter
2	Honeycombing (with or without septal thickening) involving < 25% of the lobe	Adjacent LAAs up to 10 mm in diameter
3	Honeycombing (with or without septal thickening) involving 25–49% of the lobe	LAAs > 10 mm that were adjacent to or indispensable from each other
4	Honeycombing (with or without septal thickening) involving 50–75% of the lobe	Absence of normal lung parenchyma
5	Honeycombing (with or without septal thickening) involving > 75% of the lobe	NA

LAAs, low attenuation areas

### Adverse events and response evaluation

Adverse events were graded according to the Common Terminology Criteria for Adverse Events (CTCAE), version 5.0. Management of irAEs based on AE severity following standard protocol guidelines [19]. The treating physician made all treatment strategy decisions. Since CIP is a diagnosis of exclusion, we also used criteria that were reported previously to assess the presence of CIP [20]. The patient was included in the CIP group for analyses only in cases meeting the definite criteria, patients with clinically apparent alternative diagnoses such as pulmonary infection, tumor progression, heart failure, or other etiologies were excluded. The response evaluation of PD-1 inhibitor was based on the immune-related Response Evaluation Criteria in Solid Tumors [21]. PFS was calculated from the first day of immunotherapy to the first radiological evidence of disease progression. OS was defined as the interval from the start of ICIs to the last visit or death. Censored data were defined as data from alive patients and had no evidence of disease progression at the last follow-up visit.

### Statistical analysis

Clinical characteristics, including age, gender, smoking history, tumor histology, Eastern Cooperative Oncology Group (ECOG) status, TNM stage, genetic profiles, adverse events and line of treatment were analyzed. Differences between clinic pathological characteristics was performed using the Chi-squared (Fisher’s exact test) for categorical data, and Wilcoxon rank-sum tests for continuous variables. Kaplan-Meier methodology was used to calculate median PFS and OS. All analyses were conducted using SPSS software (ver.22.0). The *p* values were two-tailed, and p values < 0.05 were considered statistically significant.

## Result

### Patient characteristics

**Fig. 1:**
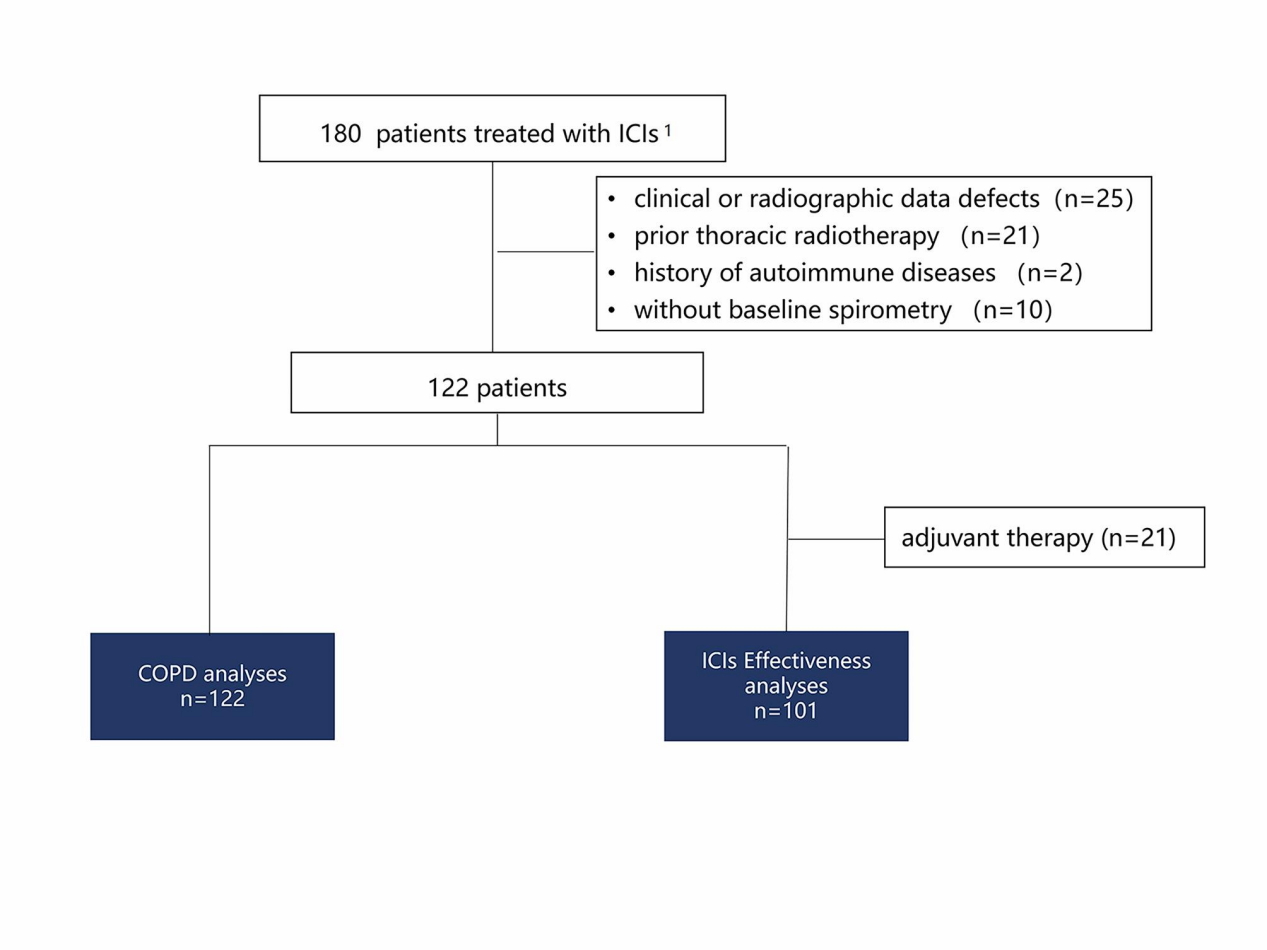
Patient flow chart of study profile. ^1^nivolumab or pembrolizumab. Abbreviations: CIP, checkpoint inhibitors related pneumonitis; COPD, chronic obstructive pulmonary disease

A total of 122 consecutive lung cancer patients were enrolled in the study (Fig. 1).

, all patients were of Chinese ethnicity. Pulmonary function tests (PFTs) are routinely performed before ICIs, starting as a screen for underlying respiratory abnormalities and baseline lung function measurements. According to the definition of chronic obstructive lung disease criteria, 50% of them (61/122) were identified as COPD.

Baseline characteristics were balanced between the lung cancer patients with and without COPD groups (Table 2). The median age of patients was 66 (range, 45–89) years old. Most patients (95.1%, 116/122) were male and had a smoking history (70.5%, 86/122). Squamous cell carcinoma (54.9%, 67/122) represents the dominant histologic subtypes, while 48 (39.4%) patients were diagnosed with adenocarcinoma lung cancer. Molecular testing, including EGFR/KRAS/NRAS/BRAF/HER-2/MET/PI3KCA mutation and ALK/ROS1/RET fusion, was performed in 41% (59/122) of patients. The expression of PD-L1 was detected by immunohistochemistry (IHC) in thirteen patients (10.7%, 13/122). Among the 13 patients whose PD-L1 expression was detected, ≥ 50% of tumor cells exhibited PD-L1 expression in were observed in 3 patients (23.0%, 3/13), 5 patients (38.5%, 5/13) with PD-L1 expression 1-49%, while 5 patients (38.5%, 5/13) with PD-L1 expression ≤ 1%.

**Table 2:** Clinical characteristics of patients (N = 122)

	Cohort (N = 122)	Groups(%)		*P*
		COPD (N = 61)	Non-COPD (N = 61)	
Age				0.230
median	66	67	66	
range	45–89	50–83	45–89	
Sex				0.680
Male	116(95.1)	59(96.7)	57(93.4)	
Female	6(4.9)	2(3.3)	4(6.6)	
Smoking history				0.073
Never-smokers	36(29.5)	13(21.3)	23(37.7)	
Ever-smokers	86(70.5)	48(78.7)	38(62.3)	
Histology				0.053
Adenocarcinoma	48(39.4)	19(31.1)	29(47.5)	
Squamous	67(54.9)	40(65.6)	27(44.3)	
Others	7(5.7)	2(3.3)	5(8.2)	
ECOG performance status				0.752
0–1	120(98.4)	60(98.4)	60(98.4)	
2–3	2(1.6)	1(1.6)	1(1.6)	
Genetic profile				0.037
EGFR	4(3.3)	1(1.6)	3(4.9)	
KRAS	7(5.7)	1(1.6)	6(9.8)	
Others	3(2.5)	1(1.6)	2(3.3)	
Negative	36(29.5)	14(23.0)	22(36.1)	
NA	72(59.0)	44(72.1)	27(44.3)	
Fibrosis score				0.638
0	100(82.0)	49(80.3)	51(83.6)	
≥1	22(18.0)	12(19.7)	10(16.4)	
Emphysema score				0.003
0	75(62.0)	29(47.5)	46(75.4)	
≥ 1	47(38.0)	32(52.5)	15(24.6)	
Baseline spirometry				0.642
DLco% predicted*	69.9(16.8)	65.9(15.2)	73.5(17.4)	
irAE				0.211
No	90(73.8)	41(67.2)	49(80.3)	
CIP	19(15.6)	11(18.0)	8(13.1)	
Other irAE	13(10.7)	9(14.8)	4(6.6)	
Treatment line				0.847
Adjuvant	21(17.2)	11(18.0)	10(16.4)	
First-line	77(63.1)	38(62.3)	39(63.9)	
Second-line	16(13.1)	7(11.5)	9(14.8)	
Subsequent-line	8(6.6)	5(8.2)	3(4.9)	

CIP, checkpoint inhibitors related pneumonitis; COPD, chronic obstructive pulmonary disease; DLco, diffusing capacity of the lung for carbon monoxide; ECOG, Eastern Cooperative Oncology Group; *EGFR*: epidermal growth factor receptor; *KRAS*, Kirsten rat sarcoma viral oncogene homolog; NA, not available; irAE, immune-related adverse events

*Values in the table represent means (standard deviation)

### Incidence of CIP in COPD patients

Generally, anti-PD-1 immunotherapy was well tolerated in lung cancer patients with COPD, the side effects are balanced in patients with and without COPD. Among the 122 patients, 19 (15.6%, 19/122) met the criteria and were diagnosed as CIP, 4 cases were grade 1, 11 were grade 2, 3 were grade 3, and 1 was grade 4. There were 11 patients with COPD who developed CIP, corresponding to 18.0% (11/61) of the COPD patients compared to 13.1% (8/61) of the non-COPD patients (*P* = 0.618). Among the 61 patients with COPD, none have spirometry-defined GOLD stage 4 (Table 3). The incidence of CIP showed no statistical difference among groups across GOLD stages (*P* = 0.796), 20% (4/20) of patients in GOLD 1 group developed CIP compared with 13.6% (3/22) in GOLD 2 group and 21.1% (4/19) in GOLD 3 group.

**Table 3:** The incidence of CIP according to GOLD stage in patients with COPD

	Total N = 61	Non-CIP (%) n = 50	CIP (%) n = 11	*P*
GOLD				0.796
1	20	16 (80.0)	4 (20.0)	
2	22	19 (86.4)	3 (13.6)	
3	19	15 (78.9)	4 (21.1)	
4	0	0 (0)	0 (0)	

Abbreviations: CIP, checkpoint inhibitors related pneumonitis; COPD, chronic obstructive pulmonary disease; GOLD, chronic obstructive lung disease

### Risk factors of CIP

The hypothesized risk factors for CIP were analyzed. In our cohort, 15.5% (19/122) patients with an F score of 1, 2.5% (3/122) patients with an F score of 2, and 82% of patients (100/122) with an F score of 0. The incidence of CIP in patients with an F score 0 was 13% (13/100). CIP occurred in 6 of 22 patients with fibrosis, higher than in patients without fibrosis in baseline CT scan (27.3% vs. 13.0%), but this difference did not reach statistical significance (P = 0.093). In contrast, in F score 1 patient with mild fibrosis and no discrete honeycombing, the incidence of CIP was 31.6% (6/19). However, no CIP occurred in F score 2 patients with honeycombing (0/3). The severity of pulmonary fibrosis score is uncorrelated with CIP (P = 0.102). Among 61 COPD patients, 12 patients with combined pulmonary fibrosis and emphysema (CPFE), the incidence of CIP in these patients was 25.0% (3/12), compared with 16.3% (8/49) in COPD patients (P = 0.676).

Emphysema, defined as an E score ≥ 1, was present in 47 patients (38.5%, 47/122). However, the presence of emphysema in the baseline did not affect the incidence of CIP (14.7% vs. 17.0%, P = 0.727). The incidence of CIP in patients with E score 0 was 14.7% (11/75), in E score 1 patient the incidence was 9.4% (3/32), and 41.7% in E score 2 (5/12). No CIP occurred in E score 3 patients (0/3), and the severity of emphysema score is uncorrelated with CIP (*P* = 0.051) either. Meanwhile, smoking history, gender, histologic tumor subtype and genetic profile did not significantly affect the risk of CIP developing.

### Treatment and outcome of patients who developed CIP

**Fig. 2:**
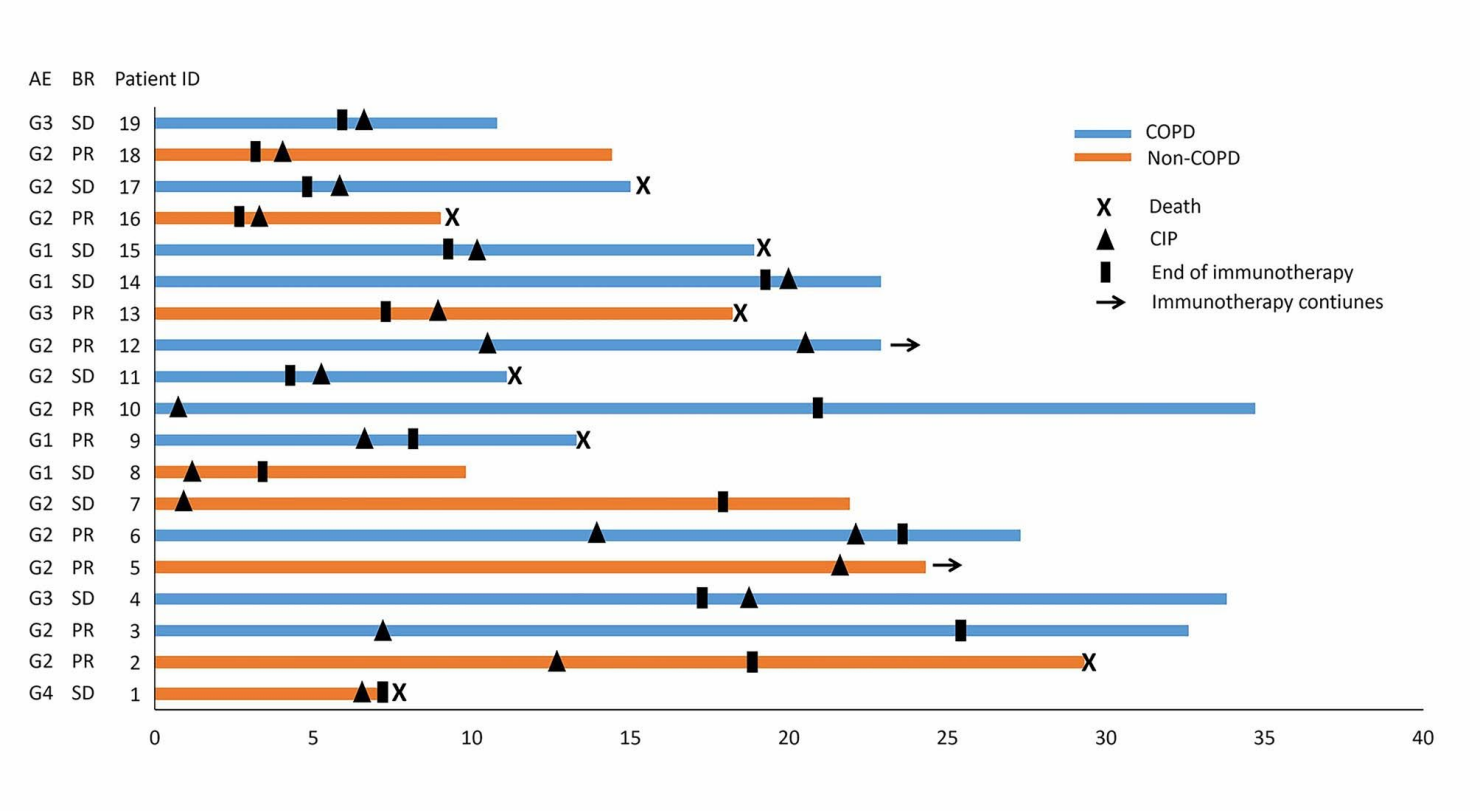
Overall survival follow-up of patients with CIP. Abbreviations: AE, adverse events; BR, best response; CIP, checkpoint inhibitors related pneumonitis; COPD, chronic obstructive pulmonary disease; G, grade of toxicity according to common terminology criteria for adverse events; PR, partial response; SD, stable disease

**Fig. 3:**
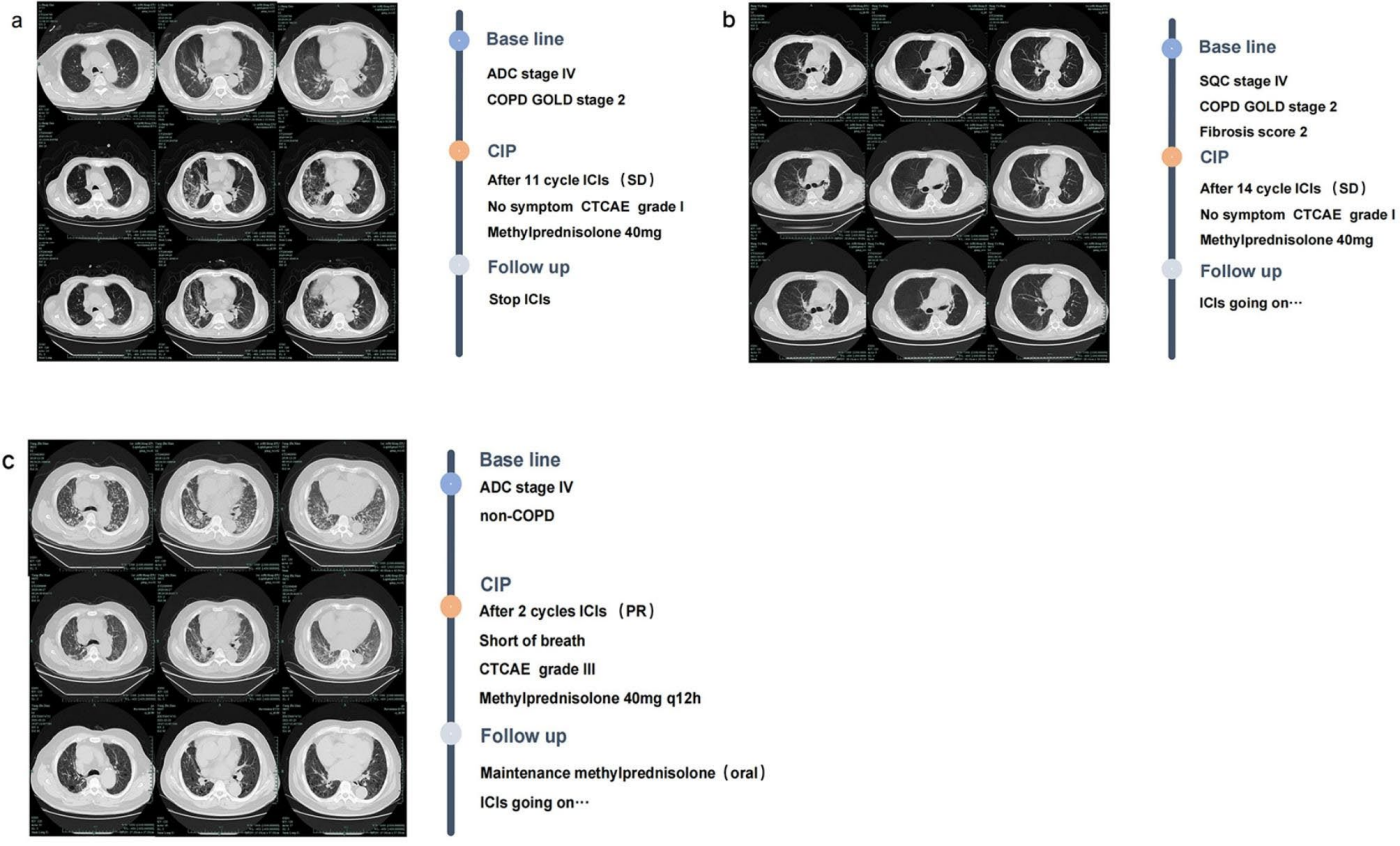
Computed tomography scan evolution of three patients developed CIP (a) patient with coexisting lung adenocarcinoma and COPD. (b) patient with pre-existing pulmonary fibrosis who presents with both lung squamous cell carcinoma and COPD. (c) lung adenocarcinoma patient without COPD. Abbreviations: ADC, adenocarcinoma; CIP, checkpoint inhibitors related pneumonitis; COPD, chronic obstructive pulmonary disease; CTCAE, common terminology criteria for adverse events, GOLD, chronic obstructive lung disease; ICI, immune checkpoint inhibitor; PR, partial response; SD, stable disease; SQC, squamous cell carcinoma

Figure 2 shows the details of the treatment regimens for CIP, CTCAE grade, and clinical courses. Among 19 patients who were observed to develop CIP, the median time to CIP diagnosis from initial ICI treatment was 6.6 months (range 0.6–22.6 months). The predominant respiratory symptoms patients complained of were shortness of breath and cough. In addition, ICIs were withheld and systemic corticosteroid therapy was initiated in 84.2% (16/19) patients as soon as CIP was diagnosed. The remaining 3 patients with CIP demonstrated only radiographic progress, yet without respiratory symptoms, were diagnosed as grade 1 and no one received adjunct treatments to corticosteroids for CIP. Among patients who received systemic corticosteroid for CIP, 93.7% (15/16) patients were improved in both imaging and symptoms after treatment, only one patient (grade 4) died 16 days later of CIP diagnosis. 36.8% of patients (7/19), all with grade 1–2 CIP, were rechallenged with ICIs for treatment of CIP, and two of them (28.6%, 2/7) showed recurrence of CIP (Fig. 2). Fortunately, the two patients with recurrence of CIP recovered on withdrawal of ICIs finally. No clear patterns emerged to differentiate the COPD-CIP from the non-COPD-CIP imaging findings. The CT images of the CIP cases are shown in Fig. 3.

### Treatment outcomes of ICIs in NSCLC patients with and without COPD

**Fig. 4:**
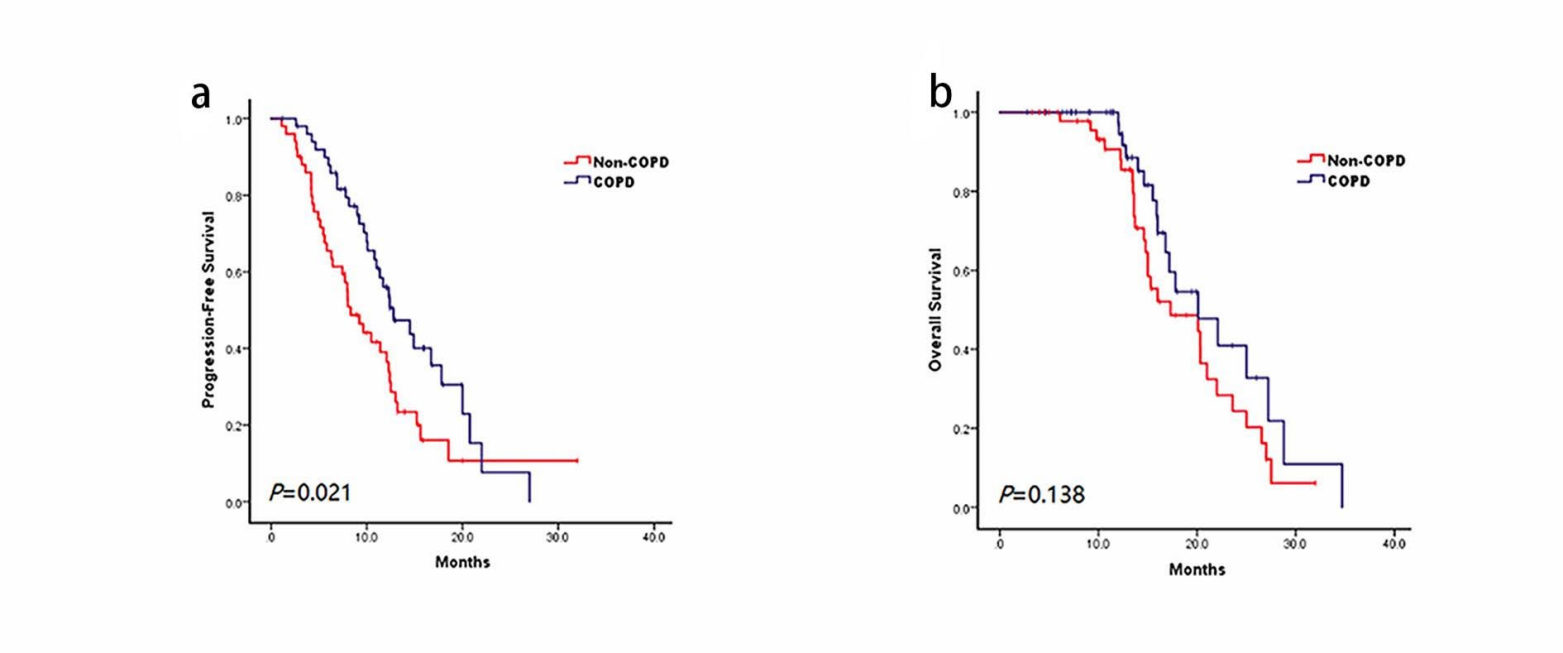
Kaplan–Meier curves of (a) progression-free survival and (b) overall survival in advanced lung cancer patients who received ICIs. Abbreviations: COPD, chronic obstructive pulmonary disease; ICI, immune checkpoint inhibitor

In the cohort, 96.7% of patients (118/122) received pembrolizumab, and the other 4 patients received nivolumab. ICIs were used predominantly in the first-line setting (63.1%, 77/122), 17.2% (21/122) received ICIs as adjuvant therapy, and the remaining 19.7% (24/122) received ICIs as second or later line immunotherapy. 87.7% of patients (107/122) received ICIs combined with chemotherapy, and single-agent anti-PD-1 immunotherapy was given to 12.3% of patients (15/122). Among these patients, 5 patients received single-agent PD-1 inhibitor as first-line therapy for their poor ECOG status or advanced age, 7 patients received single-agent as second-line therapy, and the remaining 3 patients received single-agent as subsequent treatment. Paclitaxel was an especially common agent combined with ICIs, as it was used in 92.5% (62/67) patients with squamous cell lung cancer. Whereas 75% (36/48) of patients diagnosed with adenocarcinoma received pemetrexed combined with ICIs. The survival analysis was performed on 101 patients (90%, 101/122) with metastatic NSCLC. At the time of chart extraction, 68.3% (69/101) of patients experienced disease progression. PFS was 11.4 months (95% CI = 9.2–13.5 months) for the whole cohort. Patients in the COPD group experienced a median PFS of 12.8 months (95% CI = 9.3–12.3 months) compared with only 8.3 months (95% CI = 6.2–10.5 months, *P* = 0.021) in patients without COPD (Fig. 4a). At the end of the last follow-up time, 45.5% (46/101) patients had died. The median OS was 20.1 months (95% CI = 16.6–23.6) in all study subjects. Median OS after ICI initiation was 20.1 months (95% CI = 14.4–25.8 months) for patients with COPD and 17.3 months (95% CI = 9.7–24.9 months) for patients without COPD (*P* = 0.138, Fig. 4b). Although no statistical difference was found between groups, there was a trend toward improved OS in the COPD group compared with that in a non-COPD group.

### Treatment outcomes of ICIs in COPD patients with and without CIP

**Fig. 5:**
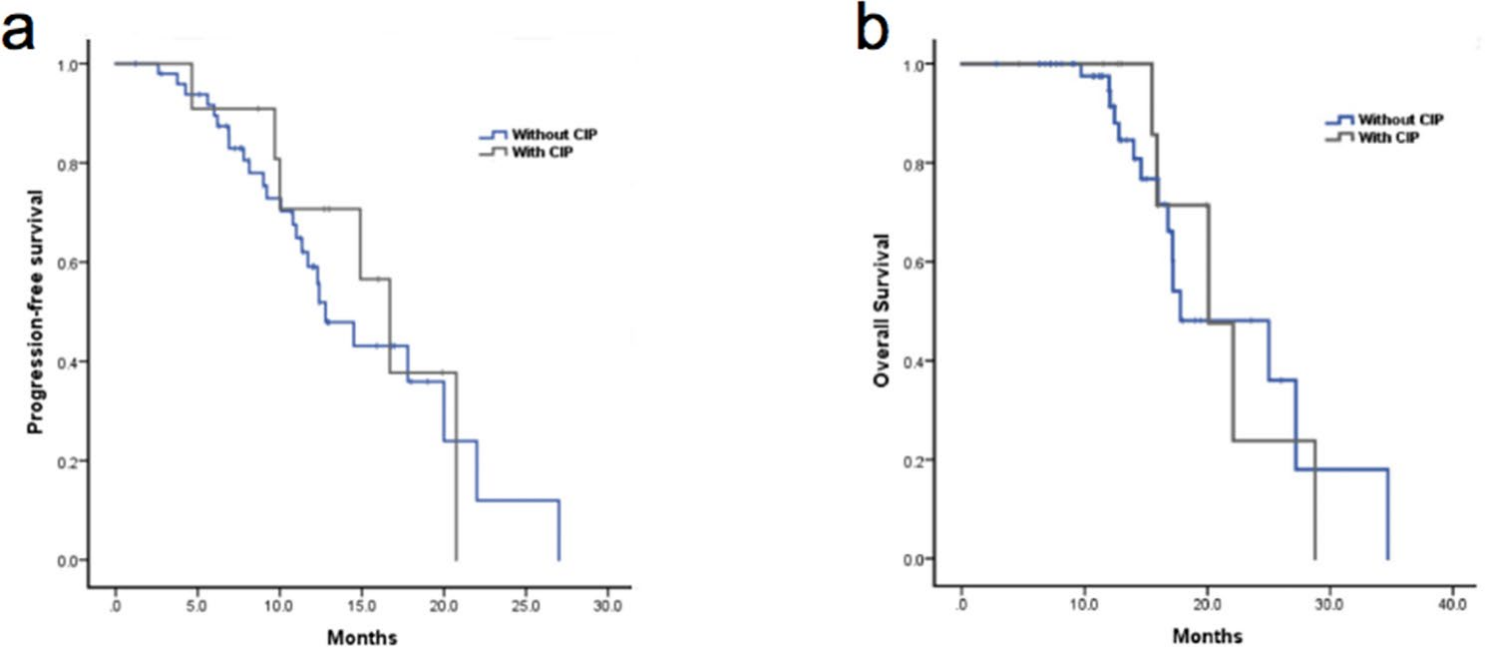
Treatment outcomes of ICIs in spirometry-defined COPD patients with and without CIP. Kaplan-Meier curve of (a) progression-free survival and (b) overall survival. Abbreviations: COPD, chronic obstructive pulmonary disease; ICI, immune checkpoint inhibitor

For patients with spirometry-defined COPD, the Kaplan-Meier curves are shown in Fig. 5. COPD patients who developed CIP were more likely to have longer PFS (16.7 vs. 12.8 months, *P* = 0.67, Fig. 5a) and OS (20.1 vs. 17.8 months, *P* = 0.90, Fig. 5b) compared with those without CIP, however the differences did not reach significance.

## Discussion

In this retrospective study, we analyzed the potential risk factors and their association with the incidence of CIP. No significant association was observed between COPD and CIP. To the best of our knowledge, this is the largest study to evaluate the impact of spirometry-defined COPD on the incidence and outcome of CIP in patients with NSCLC.

Lung cancer is the first cause of death in patients with COPD [22]. COPD is a disease associated with chronic inflammation of the airways and lung parenchyma, characterized by persistent lung injury, activates a regulatory mechanism that downgrades the immune response, including PD-L1/PD-1 [23], and thus opening a therapeutic window among patients with COPD who develop lung cancer. Despite the proven longer PFS in lung cancer patients with COPD [24–26], the uncertain safety may limit the use of ICIs. Since it is biologically plausible for immunotherapy to stimulate lymphocytes against healthy lung cancer cells, increasing lung tissue damage and COPD symptoms. Nair et al. reported a series of patients with prolonged and severe COPD exacerbations upon initiating immune checkpoint inhibitor therapy, indicating ICIs may aggravate CODP and irAE [27]. However, given the stringent eligibility criteria applied in clinical trials, healthier patients tend to be enrolled, and thus the real-world frequency of these events in the overall population is unknown. Findings from three recent studies indicated that COPD might associate with CIP [20, 28, 29]. However, these studies have not accounted for some crucial potential confounding factors. Atchley et al. [20] showed that obstructive lung disease was independently associated with CIP (aOR, 2.79; 95% CI, 1.07–7.29). It is noteworthy that the history of COPD is not a statistically significant risk factor for CIP in multivariate logistic regression analysis. Moreover, only 24.1% of these patients had documented baseline spirometry. Additionally, patients with ILD, connective tissue disease (CTD), and prior thoracic radiation history were included in the above research for CIP analysis, while the clinical variables mentioned above have been demonstrated as an independent risk factors for the development of CIP [6, 30, 31]. All the mentioned interfering factors above are difficult to distinguish from the risk conferred by COPD. Another study by Sul et al. [28] recapitulated this finding that CIP occurred more frequently in patients with a history of COPD and asthma than in patients without this history (5.4% vs. 3.1%). Unfortunately, in the study COPD and asthma were grouped together, which could be inappropriate given that the pathophysiology and dominant immunologic mechanisms involved differ [32]. A recent retrospective study reported that the presence of COPD was independently associated with a higher incidence of CIP, 70% of the patients in the CIP group had COPD. Unfortunately, the study investigated this association using physician-diagnosed COPD [29].

Notably, the incidence of CIP was similar in patients regardless of COPD status (18.0% vs. 13.1%, *P* = 0.618) in our study. The discrepancy might be partly attributed to the confounding factors in the previous study, and therefore, conclusions from these studies to be addressed with caution. Overcoming these limitations, the present study firstly showed that coexisting spirometry-defined COPD was not an independent risk factor for patients with lung cancer who have co-morbid COPD. In our study, the incidence of all CIP was 15.5% (19/122), and the incidence for grade ≥ 3 CIP was 3.3% (4/122), which is consistent with a prior report [6]. With timely and appropriate systemic corticosteroid treatment, the clinical symptoms and imaging changes of CIP can be improved in most patients (93.8%, 15/16). These results support the clinical observation that lung cancer patients can be treated safely with ICI in the context of COPD, and treatment-related irAEs were found to be manageable in COPD patients.

Pre-existing ILD has been reported to exist in approximately 15% of lung cancer patients at the time of initial diagnosis and is associated with a poor prognosis [33, 34]. Since patients with pre-existing pulmonary fibrosis are often excluded in clinical trials of immunotherapy to avoid CIP, the relationship between them needs to be further elucidated. Interstitial lung abnormalities (ILA) are defined as increased lung densities on chest computed tomography images of patients without previous history of ILD [35]. In our retrospective study, analysis according to the F score on baseline CT was analyzed in patients with ILA. The incidence of CIP was similar in two arms, which is inconsistent with previous studies [9, 11, 17]. This may partially attribute to the small sample size of patients with pre-existing pulmonary fibrosis in our study, and thus could not detect the group differences. Additionally, consideration is given to potential harms in the individual patients, physician may be more cautious to prescribe ICIs to patients with severe CT abnormalities (fibrosis/emphysema) in clinical practice. In our study, only 3 patients with F score 2 (presence of honeycomb lungs) received ICIs. According to the result of a phase 2 trial of atezolizumab for pretreated NSCLC with idiopathic interstitial pneumonitis, there is a particularly high risk of CIP in patients with a honeycomb lung on HRCT [11].

We also compared other clinical variables such as smoking history, older age, poor performance status, and underlying medical comorbidities, considered high-risk factors for drug-induced ILD [9, 30, 36]. No significant association was observed between groups according to the variables mentioned above.

Previous study indicated that some patients didn’t meet the spirometric standard of COPD have evidence of structural lung disease (emphysema, gas trapping) on chest imaging, these patients may experience a high risk of lung function decline and have potential to develop COPD in the future [37]. Previous studies reported that pre-existing pulmonary emphysema on baseline chest CT is not a risk factor for CIP [17, 20]. In our study, 24.6% (15/61) of patients in non-COPD group with emphysema in baseline CT scan, we also analyzed the relationship between E score and CIP. Consistent with previous studies, no relation between E score and CIP was revealed in our study.

Several retrospective studies suggest a longer PFS to ICIs in NSCLC patients with COPD compared to those without COPD [24, 26, 38]. However, there was also a limitation in the studies, only a small proportion of patients had documented spirometry in baseline and investigated COPD using physician-diagnosed COPD [24, 26]. In our study, we confirmed the conclusion in spirometry-defined COPD patients with co-morbid lung cancer. Moreover, the findings of previous studies were inconsistent regarding OS. A retrospective study in China was found a prolonged OS in the subgroup of patients with mixed ventilatory defects [26]. Nevertheless, numerous diseases are causing mixed ventilatory pulmonary, and the pathophysiology and underlying molecular mechanisms are different. Therefore, the difference in OS between groups cannot solely be attributed to COPD and the conclusion of the study should be treated with caution. Previous studies demonstrated patients with irAE, especially with lower-grade irAE was associated with better outcomes in patients with ICI treatment for NSCLC [39, 40]. In this study, 78.9% patients (15/19) with grade 1 or 2 CIP. Likewise, we observed that patients with CIP had longer median PFS and OS versus those without CIP in COPD subgroup, but the difference was not statistically significant given the limited sample size.

There are several limitations to this study, which should be addressed. First, the retrospective nature of this study is prone to biases from missing data and reliance on the documentation available for review. Second, the number of patients with CIP might not be sufficient to provide reliable information concerning the significance of the subgroup analysis according to COPD severity. Third, due to the scarcity of lung tissue from patients who received an ICIs, we lacked data regarding the PD-L1 expression and tumor mutational burden (TMB) of patients with COPD, unable to explore the potential interaction between COPD and immune profile. However, our data may be more informative in a real-world clinical setting, as clinical trials have demonstrated that patients achieved clinical benefit from ICI combined chemotherapy irrespective of PD-L1 expression [41], and patients who undergo palliative immunotherapy for NSCLC do not routinely undergo PD-L1 or TMB detection. Further prospective studies are needed to elucidate the complex relationship between the immune profile in tumor and the incidence of CIP in the context of COPD.

## Conclusion

The finding of our study identifying coexisting COPD did not predict the higher risk of CIP, and supports the clinical observation that lung cancer patients can be treated safely with ICIs in the context of COPD. Although there is no statistic difference, an increasing trend in the incidence of CIP among patients with pulmonary fibrosis on baseline chest CT scans. Moreover, we confirmed that COPD was associated with a longer PFS to ICI treatment, by using an objective measure for COPD diagnosis in NSCLC patients. This study fills an essential gap in the literature and supplements the limited number of published reports on CIP in the context of COPD. Further clinical studies are warranted to validate these findings.

## Data Availability

The datasets generated and analyzed during the current study are available from the corresponding author on reasonable request.
